# Primary Soft-Tissue Hydatid Disease of the Posterior Thoracic Wall: A Rare Presentation

**DOI:** 10.4269/ajtmh.26-0262

**Published:** 2026-06-09

**Authors:** Laila Jedidi, Anis Kerkeni, Senda Ben Lahouel

**Affiliations:** ^1^ Department of General Surgery, Jendouba Regional Hospital, Jendouba, Tunisia;; ^2^ Faculty of Medicine of Tunis, University of Tunis El Manar, Tunis, Tunisia;; ^3^ Department of General Surgery, Sidi Bouzid Regional Hospital, Sidi Bouzid, Tunisia;; ^4^ Faculty of Medicine of Sousse, University of Sousse, Sousse, Tunisia

## CASE PRESENTATION

**Figure 1.**
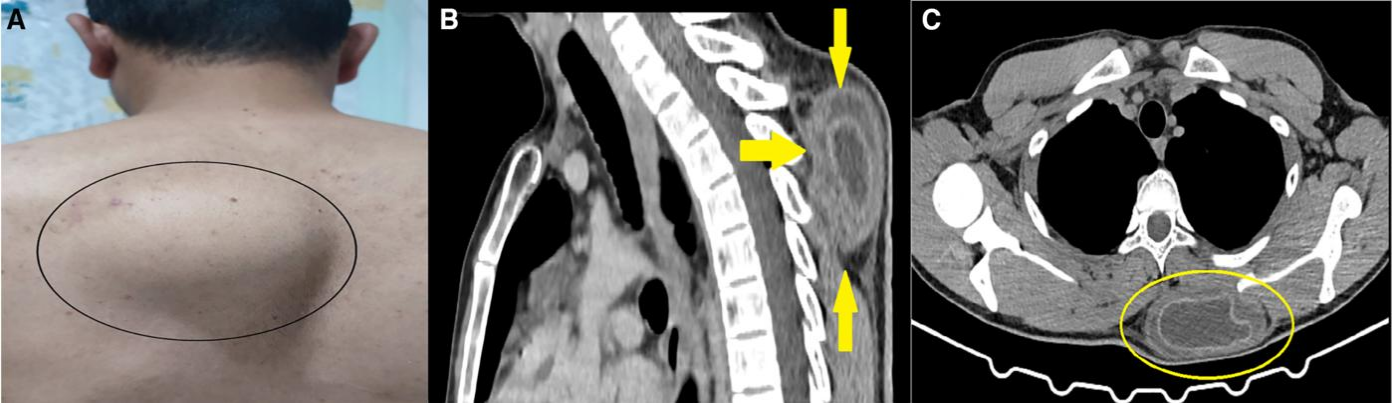
(**A**) Physical examination of a mass in the left scapular region (black circle). (**B**) Sagittal computed tomography (CT) image revealing a well-defined cystic lesion of the posterior thoracic wall (yellow arrows). (**C**) Axial CT image revealing the superior portion of the cystic mass (yellow circle), confirming its subcutaneous location measuring ∼9 cm. The mass is confined to the subcutaneous soft tissues without invasion of underlying muscles or bone.

A 25-year-old male patient with no significant medical history presented with a slowly growing mass in the left scapular region, which he had first noticed 5 years earlier but had ignored since then. A physical examination revealed a painless, subcutaneous cystic mass measuring ∼10 cm, without signs of inflammation or pus discharge (Figure 1A). Computed tomography revealed a well-circumscribed, oval cystic lesion with a thick but regular wall located within the subcutaneous soft tissues of the posterior thoracic wall, adjacent to the D3 to D5 vertebral levels, in the left paramedian region (Figure 1B). The mass was situated superficial to the latissimus dorsi muscle and measured 9 × 5 × 9 cm (Figure 1C). No pulmonary or hepatic cysts were identified. Hydatid disease was suspected, and serology results via ELISA were positive. Magnetic resonance imaging further supported the diagnosis of a subcutaneous hydatid cyst of the left posterior thoracic wall.

**Figure 2.**
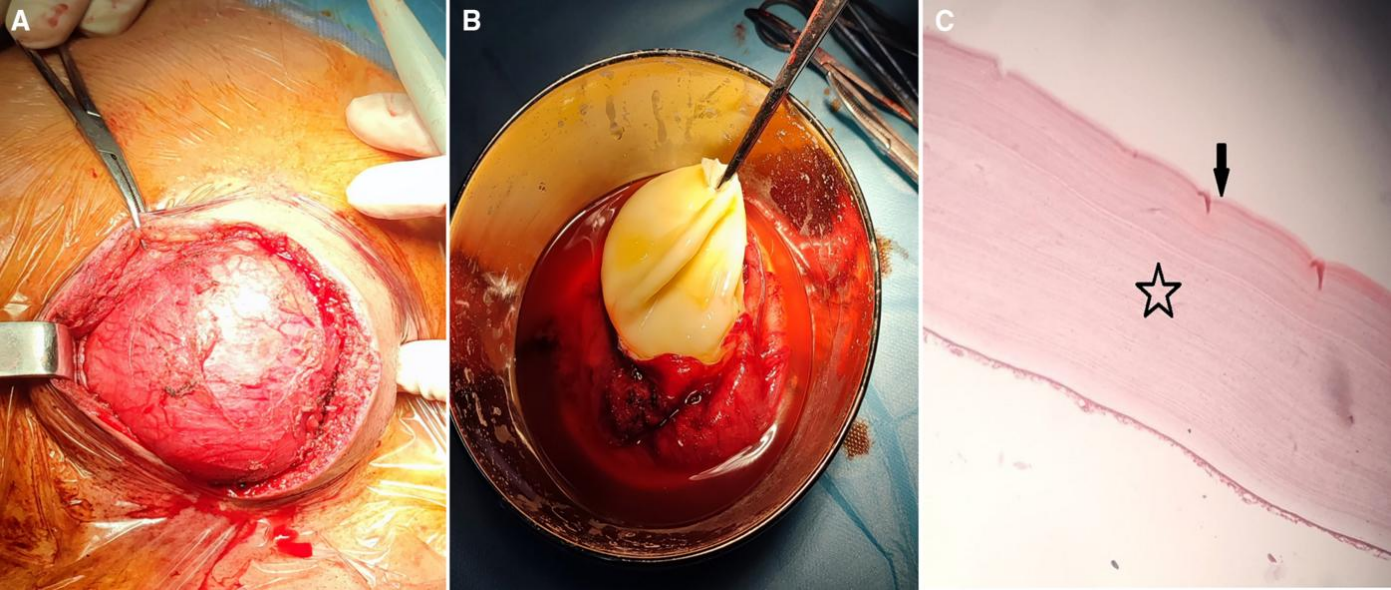
(**A**) Intraoperative image revealing the subcutaneous cyst. (**B**) Extraction of the germinative membrane from the cyst cavity after opening the cyst. (**C**) Histological section of the hydatid cyst revealing the cyst wall with an inner germinal layer (black arrow) and a laminated membrane (black star; hematoxylin and eosin ×40).

The patient underwent surgery with complete excision of the cyst (Figure 2A and B). The postoperative course was uneventful. Albendazole therapy was prescribed for two cycles of 28 days separated by a 14-day interval to reduce the risk of recurrence. A histopathological examination confirmed the diagnosis of a hydatid cyst (Figure 2C).

Subcutaneous hydatid cysts represent an exceptionally rare manifestation of *Echinococcus granulosus* infection, accounting for less than 2% of all hydatid disease localizations.^1^ Their occurrence without concomitant hepatic or pulmonary involvement is more uncommon because the liver and lungs typically act as the first and second filtration barriers for the parasite.^2^ The unusual presentation in the patient, with a solitary cyst confined to the posterior thoracic subcutaneous tissues, highlights the atypical nature of this localization.

The exact mechanism underlying subcutaneous involvement remains poorly understood.^3^ This rarity often leads to delayed diagnosis because soft-tissue hydatid cysts can mimic other benign cystic or tumoral lesions.^4^ Complete surgical excision followed by anthelminthic therapy is the optimal treatment to minimize postoperative recurrence.^5^
